# Supplementary material for: The implementation of prioritization exercises in the development and update of health practice guidelines: A scoping review
Source: PLoS One. 2020 Mar 20;15(3):e0229249. doi: 10.1371/journal.pone.0229249 (PMC7083273; doi:10.1371/journal.pone.0229249)
Supplement: S2 File — (DOCX) [file pone.0229249.s002.docx]

# Study Protocol

**Prioritization approaches, tools and processes in health: a scoping review**

**Keywords:** priority setting, prioritization approaches, health priorities, research prioritization, methodology, systematic review

**Ethical approval:** The study did not involve human subjects thus did not require ethical approval.

**Background**

Priority setting exercises in healthcare can have slightly different nuances based on the level they are focused: research, policy or interventional.

The academic world has defined research priorities as the “explicit areas of agreement”, among the overlapping interests of actors and the ranking process of these priorities across the different actors’ research agendas (Gonzalez-Block, 2004). Research priority exercise aims at promoting collaboration between research entities and reducing the duplication of effort required to set priorities with the highest number of health benefits to populations (Mador, Kornas, Simard, & Haroun, 2016). The consensus on a set of research agendas can then be used as a policy instrument, or a facilitator for its development, to achieve a common end that urgently requires an amendment without enforcing a single research agenda while doing so (Mador et al., 2016; World Health Organization, 2012).

Policy makers adopt priority setting exercises to rank health priorities thus allocating the funds appropriately among competing elements of the healthcare system, especially when the resources available are scarce (Byskov et al., 2014; Guindo et al., 2012).

The same applies for setting priorities for interventional programs. The WHO group defines several components to choosing the number one priority intervention. The process consists of three major steps: Data (evidence), Dialogue (transparency) and Decision (voice). The first step (data) consists of several criteria: burden of the health issue, effectiveness, cost and acceptability of the intervention. In the second step, participatory policy dialogues take place in which criteria can be challenged and has the potential for improvement. In the last step, decision making and validation can be voiced by either the parliament, cabinet, laws and decrees, citizen’s jury or a national health assembly (World Health Organization, 2010).

Despite the minor nuances in the process of priority setting exercises across the three levels, there is an agreement that it is an iterative and interactive consensus forum, in which stakeholders meet to negotiate health planning options that aim to increase the welfare of its population. In other terms, health care decision making directs stakeholders towards balancing between the demand for essential needs and the services that enhance human health, economic restraint and scarce resource pool (Cromwell, Peacock, & Mitton, 2015; Okello & Chongtrakul, 2000). However, priorities differ from one government and its individuals to another, and is defined by their capacity, purposes, mandate, culture and resources. Many studies reported that the most important aspect of priority setting is to ask the question of “Priorities for whom?” and to set explicit criteria to guide a transparent and consistent decision making process (Cromwell et al., 2015; Guindo et al., 2012; Okello & Chongtrakul, 2000).

A priority setting exercise may differ according to the approaches used. Some of these approaches are: the Delphi method, the nominal group technique (NGT), Essential National Health Research (ENHR), The Council on Health Research and development (COHRED), The Child Health and Nutrition Research Initiative (CHNRI), the three-Dimensional Comprehensive Approaches Matrix (3D CAM), Stepwise process, the consensus development conference, Programme Budgeting and Marginal Analysis (PBMA) and Multi-Criteria Decision Analysis (MCDA). These tools facilitate the collection, organization and analysis of information needed to aid setting priorities (Ghaffar, 2009).

According to Clavisi et al, priority setting is essential to make the best choices about the funding for health services and treatments, research, health technology assessments, guideline development, and development of systematic reviews (Clavisi, Bragge, Tavender, Turner, & Gruen, 2013). From an economic perspective, these exercises allow decision makers to adopt an opportunity cost approach. Meaning, decision makers choose between a minimum of two set of options by allocating funds to only one while taking into account their scarce resources (Mitton & Donaldson, 2003). In addition, these exercises, if correctly used with an explicit framework of action, criteria and appropriate evaluation, will increase transparency and accountability of these choices on all levels of priority setting (Baltussen & Niessen, 2006; Kapiriri & Martin, 2010). Priority setting exercises are an essential and meaningful first step in consensus building, especially in low- and middle-income countries (LMICs), given the limited resources and the large number of issues to be tackled (Bigdeli, Javadi, Hoebert, Laing, & Ranson, 2013). This initial step might be considered lengthy for decision makers but while the population’s health is at stake, some time spent on resource allocation is deemed crucial. The opportunity cost gained from such exercises is a great factor to be considered for the ultimate priority setting environment (Mitton & Donaldson, 2009).

Priority setting exercises are considered to be essential requirements in all health care systems worldwide. There is a clear need, however, to find a standardized definition of what constitutes a successful priority setting exercise in order to offer a common language and agreement on conceptual basis for this concept (Sibbald, Singer, Upshur, & Martin, 2009). These exercises happen constantly whether the appropriate tool was used and explicitly reported. Although it has been described as a “value laden”, political and a difficult decision making procedure, few are trying to develop the theories behind these tools and their validation (Rudan et al., 2010). In addition to being value laden, the process is considered very technical encompassing burden of disease data, cost-effectiveness analyses, clinical trials, among others, all the latter do not prepare and equip decision makers to tackle wider societal values such as equity, trust, accountability and fairness (Byskov et al., 2009). Moreover, decision makers sometimes are not aware of the availability of these tools and lack guidance on how to use those (Sibbald et al., 2009). This raised the issue of trust and credibility in resource allocation decision making and pushed leaders to desire an explicit framework to guide priority setting exercises (Mitton & Donaldson, 2009; Sibbald et al., 2009). Hence the need of a standardized and validated priority setting tools for smoother, comprehensive and replicable procedures.

**Objectives**

To identify priority setting exercises that have been conducted, and approaches and tools that have been utilized or proposed to set priorities in the health field.

**Methods**

*Eligibility criteria:*

- Study type: all study designs except for editorials, commentaries, correspondences, letters, news, abstracts, or reviews.
- Scope:
  - We will include papers describing at least one of the following: the development, validation, implementation, or evaluation of prioritization approaches, exercises/processes or tools. We will focus on the clinical, public health and health systems fields.
  - We will exclude papers describing individual prioritization criteria (e.g. authors list criteria for prioritization such as burden of disease and cost, but do not describe a prioritization approach or exercise/process).
  - We will exclude papers that describe research priorities but do not provide sufficient details about the process or methodology (e.g. authors list priority topics without describing how they identified them).
- Subject of the prioritization: any type of research, including primary research, evidence syntheses (e.g. systematic reviews), and practice guidelines.
- Setting: We will include eligible papers irrespective of whether the setting was low-, middle- or high-income countries, or primary, secondary or tertiary healthcare facilities.

*Information sources:*

- We will search MEDLINE and CINAHL electronic databases. We will search each database by combining two different concepts: “priority setting exercise” and “tools”. The search terms that will be used to search the different databases are found in Appendix 2. The search will include free text-words and MeSH terms. We will also manually search key databases such as Cochrane Library and Google Scholar.
- We will use no language or year of publication restrictions.
- We will develop the search strategy with an information specialist at the American University of Beirut.
- We will screen the reference list of included studies.

*Study selection:*

Study selection will be completed in two phases:

- Title and abstract screening: Teams of two reviewers will use the above eligibility criteria to screen titles and abstracts duplicate and independently. If a paper describes a topic that fits more than one category (public health, clinical practices and/or health systems), it will be added to all relevant categories. We will get the full text of citations judged as potentially relevant by at least one of the reviewers in each team.
- Full-text screening: Teams of two reviewers will use the same eligibility criteria to screen the full texts of studies in duplicate and independently for eligibility. They will resolve disagreements by discussion or with the help of a third reviewer when consensus cannot be reached.
- We will use a standardized and pilot-tested screening forms.
- We will also conduct calibration exercises to enhance the validity of the screening process.

*Data abstraction:*

- Teams of two reviewers will abstract data from eligible studies in duplicate and independently. They will resolve disagreement by discussion or with the help of a third reviewer when consensus cannot be reached.
- We will collect the following data:
- Study information (authors, year of publication);
- Subject of the prioritization: primary research vs. evidence syntheses vs. practice guidelines;
- Paper reports on conducted priority setting exercises (i.e. process) vs. priority setting approaches/tools;
- Field (clinical, public health, health systems research)
- Development and/or validation and/or implementation and/or evaluation;
- Description of the priority setting approach/tool or exercise/process (e.g. steps involved, stakeholders, criteria);
- Information on the approach/tool or exercise/process: name and items;
- Target user of the prioritization process or tool (policymakers, researchers etc.).
- We will use standardized and pilot-tested data abstraction forms.
- We will conduct calibration exercises to enhance the validity of the data abstraction process.

*Data synthesis:*

We will use a descriptive approach for the synthesis and presentation of information on the general characteristics of the studies. The results generated will be presented in a tabular form. The tables will present an overview of the included studies.

**References**

Baltussen, R., & Niessen, L. (2006). Priority setting of health interventions: the need for multi-criteria decision analysis. *Cost Effectiveness and Resource Allocation, 4*(1), 14.

Bigdeli, M., Javadi, D., Hoebert, J., Laing, R., & Ranson, K. (2013). Health policy and systems research in access to medicines: a prioritized agenda for low-and middle-income countries. *Health Research Policy and Systems, 11*(1), 37.

Byskov, J., Bloch, P., Blystad, A., Hurtig, A.-K., Fylkesnes, K., Kamuzora, P., . . . Martin, D. K. (2009). Accountable priority setting for trust in health systems-the need for research into a new approach for strengthening sustainable health action in developing countries. *Health Research Policy and Systems, 7*(1), 23.

Byskov, J., Marchal, B., Maluka, S., Zulu, J. M., Bukachi, S. A., Hurtig, A.-K., . . . Nyandieka, L. N. (2014). The accountability for reasonableness approach to guide priority setting in health systems within limited resources–findings from action research at district level in Kenya, Tanzania, and Zambia. *Health Research Policy and Systems, 12*(1), 49.

Clavisi, O., Bragge, P., Tavender, E., Turner, T., & Gruen, R. L. (2013). Effective stakeholder participation in setting research priorities using a Global Evidence Mapping approach. *J Clin Epidemiol, 66*(5), 496-502. e492.

Cromwell, I., Peacock, S. J., & Mitton, C. (2015). ‘Real-world’health care priority setting using explicit decision criteria: a systematic review of the literature. *BMC Health Services Research, 15*(1), 164.

Ghaffar, A. (2009). Setting research priorities by applying the combined approach matrix. *Indian Journal of Medical Research, 129*(4), 368.

Gonzalez-Block, M. A. (2004). Health policy and systems research agendas in developing countries. *Health Research Policy and Systems, 2*(1), 6. doi: 10.1186/1478-4505-2-6

Guindo, L. A., Wagner, M., Baltussen, R., Rindress, D., van Til, J., Kind, P., & Goetghebeur, M. M. (2012). From efficacy to equity: Literature review of decision criteria for resource allocation and healthcare decisionmaking. *Cost Effectiveness and Resource Allocation, 10*(1), 9.

Kapiriri, L., & Martin, D. K. (2010). Successful priority setting in low and middle income countries: a framework for evaluation. *Health Care Analysis, 18*(2), 129-147.

Mador, R. L., Kornas, K., Simard, A., & Haroun, V. (2016). Using the Nine Common Themes of Good Practice checklist as a tool for evaluating the research priority setting process of a provincial research and program evaluation program. *Health Research Policy and Systems, 14*(1), 22. doi: 10.1186/s12961-016-0092-5

Mitton, C., & Donaldson, C. (2003). Resource allocation in health care: health economics and beyond. *Health Care Analysis, 11*(3), 245-257.

Mitton, C., & Donaldson, C. (2009). *Priority setting toolkit: guide to the use of economics in healthcare decision making*: John Wiley & Sons.

Okello, D., & Chongtrakul, P. (2000). *A manual for research priority setting using the ENHR strategy*: COHRED.

Rudan, I., Kapiriri, L., Tomlinson, M., Balliet, M., Cohen, B., & Chopra, M. (2010). Evidence-based priority setting for health care and research: tools to support policy in maternal, neonatal, and child health in Africa. *PLoS medicine, 7*(7), e1000308.

Sibbald, S. L., Singer, P. A., Upshur, R., & Martin, D. K. (2009). Priority setting: what constitutes success? A conceptual framework for successful priority setting. *BMC Health Services Research, 9*(1), 43.

World Health Organization. (2010). Priority interventions: HIV/AIDS prevention, treatment and care in the health sector.

World Health Organization. (2012). *The WHO strategy on research for health*: World Health Organization.
