# Supplementary material for: The implementation of prioritization exercises in the development and update of health practice guidelines: A scoping review
Source: PLoS One. 2020 Mar 20;15(3):e0229249. doi: 10.1371/journal.pone.0229249 (PMC7083273; doi:10.1371/journal.pone.0229249)
Supplement: S3 File — (DOCX) [file pone.0229249.s003.docx]

# **Supplementary file 3:** Search strategy

**Databases Searched:**

| **Database** | **Number of hits** |
| --- | --- |
| Medline | 22,022 |
| CINAHL | 11,217 |
| Google Scholar | 100 |
| **Total** | **33,339** |

**Search Strategy for each database:**

**Database: Ovid MEDLINE(R)** Epub Ahead of Print, In-Process & Other Non-Indexed Citations, Ovid MEDLINE(R) Daily and Ovid MEDLINE(R) <1946 to June 2017>

Search Strategy:

--------------------------------------------------------------------------------

1 Methods/

2 (framework or frameworks or approach or approaches or tool or tools or checklist or checklists or strategy or strategies or process or processes or criteria or toolkit or technique or techniques).ti,ab.

3 1 or 2

4 Resource Allocation/

5 health planning guidelines/ or health planning technical assistance/ or health priorities/ or regional health planning/ or health systems plans/ or community health planning/

6 health care rationing/

7 ("health planning" or "health priorit*" or "health systems plans " or "health care rationing" or "resource allocation").tw.

8 ((framework or frameworks or approach or approaches or tool or tools or checklist or checklists or process or processes or criteria or toolkit or technique or techniques) adj5 (priorit* or agenda? or (resource adj allocation))).ti,ab.

9 4 or 5 or 6 or 7

10 3 and 9

11 8 or 10

***************************

**Database: CINAHL**

Search Strategy:

--------------------------------------------------------------------------------

S1

MJ methods OR TI ((framework or frameworks or approach or approaches or tool or tools or checklist or checklists or strategy or strategies or process or processes or criteria or toolkit or technique or techniques)) OR AB ((framework or frameworks or approach or approaches or tool or tools or checklist or checklists or strategy or strategies or process or processes or criteria or toolkit or technique or techniques))

S2

MJ ( “Resource Allocation” OR “health planning guidelines” or “health planning technical assistance” or “health priorities” or “regional health planning” or “health systems plans” or “community health planning” OR “health care rationing” ) OR TX ( ("health planning" or "health priorit*" or "health systems plans " or "health care rationing" or "resource allocation") )

S3 (S1 AND S2)

S4

TI (((framework or frameworks or approach or approaches or tool or tools or checklist or checklists or process or processes or criteria or toolkit or technique or techniques) N5 (priorit* or agenda? or (resource N1 allocation)))) OR AB (((framework or frameworks or approach or approaches or tool or tools or checklist or checklists or process or processes or criteria or toolkit or technique or techniques) N5 (priorit* or agenda? or (resource N1 allocation))))

S5 (S3 OR S4)

***************************

**Google Scholar**

Last searched: July 2019

(method|framework|approach|tool|checklist|strategy|strategies|process|criteria|toolkit|technique) (health planning|health priority|health priorities|health prioritization|health systems plans|health care rationing|agenda|resource allocation)

*The 1^st^ 100 articles were retrieved
