# Supplementary material for: The implementation of prioritization exercises in the development and update of health practice guidelines: A scoping review
Source: PLoS One. 2020 Mar 20;15(3):e0229249. doi: 10.1371/journal.pone.0229249 (PMC7083273; doi:10.1371/journal.pone.0229249)
Supplement: S4 File — (DOCX) [file pone.0229249.s004.docx]

**Supplementary file 4: List of data extraction variables**

*General information*

- Author's last name and date of publication
- Lead entity: entity that initiated the prioritization
- Aim of study
- Target audience
- Year of prioritization conduct
- Scope of prioritization
- Topic of prioritization (clinical, public health, health systems) and specific domain
- Focus of prioritization (topics, questions, outcomes)
- Type of guideline development (de novo development, update, adaptation)
- Description of conducted prioritization exercise

*Prioritization steps*

- Principles guiding prioritization
- Generation of initial list of topics (identification of existing trials; identification of existing systematic reviews; identification of existing guidelines; build on data from health information system; build on previous priority setting exercises; stakeholder input)
- Collection of technical data (e.g., burden of disease or cost-effectiveness data)
- Use of established prioritization methods (e.g., James Lind Alliance methodology)
- Research gap analysis (i.e., mapping of evidence against priority topics)
- Criteria-related factors
  - Use of prioritization criteria
  - Ranking of priorities (consensus versus metrics)
- Post-prioritization
  - Refinement of priorities into guideline topics
  - Dissemination and implementation
  - Revision mechanism
  - Monitoring and evaluation

*Stakeholder input*

- - Type(s) of stakeholders (we used the 7Ps framework)
  - Recruitment method
  - Method(s) of engagement

*Prioritization outputs*

- - Type of output (e.g., topics, questions)
  - Presentation of outputs (e.g., ranked or non-ranked)
