# Supplementary material for: The implementation of prioritization exercises in the development and update of health practice guidelines: A scoping review
Source: PLoS One. 2020 Mar 20;15(3):e0229249. doi: 10.1371/journal.pone.0229249 (PMC7083273; doi:10.1371/journal.pone.0229249)
Supplement: S5 File — (DOCX) [file pone.0229249.s005.docx]

**Supplementary file 5: Prioritization criteria framework**

| Problem- or disease-related considerations | Health burden |
| --- | --- |
|  | Economic burden |
|  | Burden on healthcare system |
|  | Equity relevance |
|  | Urgency |
| Interest | Health professional level |
|  | Consumer level |
|  | National level |
| Practice considerations | Practice variation |
|  | Uncertainty or controversy about best practice |
| Guideline development | Absence of guidance |
|  | Unsatisfactory guidance |
|  | Availability of evidence |
|  | Availability of low certainty evidence |
|  | Potential for changing existing guidance |
| Potential impact of the intervention | Health policy and practice |
|  | Impact on health outcomes |
|  | Economic impact |
|  | Impact on the healthcare system |
|  | Impact on equity/access |
| Implementation considerations | Feasibility of intervention implementation |
|  | Availability of resources |
|  | Applicability |

| Prioritizing de novo development of guidelines | |
| --- | --- |
| Authors’ prioritization criteria | **Prioritization criteria reclassification** |
| Loeffen, 2015 (3 criteria) | |
| prevalence of each supportive care topic | Health burden |
| severity of each supportive care topic | Health burden |
| adequate treatment options for each supportive care topic | Impact on health outcomes |
| Nast, 2019 (7 criteria) | |
| feasibility of a systematic search for each topic | Judged by authors as irrelevant |
| health burden on the population | Health burden |
| economic burden of disease on society | Economic burden |
| cost of treatment to the health care system | Burden on healthcare system |
| extent of variation in practice | Practice variation |
| state of scientific knowledge | Potential for changing existing guidance |
| cost of guideline development | Judged by authors as irrelevant |
| Kerr, 2009 | |
| *No explicit criteria used* | |
| Brouwers, 2003 (4 criteria) | |
| relevance of a guideline on the subject to the practitioner's practice | Interest at health professional level |
| proportion of clinical cases of the practitioners involving anticonvulsant use (topic being prioritized) | Health burden |
| knowledge of existing guidelines on this topic | Judged by authors as irrelevant |
| interest in reviewing a completed practice guideline and three clinical scenarios | Judged by authors as irrelevant |
| Farrell, 2015 (10 criteria) | |
| weighing benefits vs. harms of medication therapy (e.g. where harm of continuing the medication, such as actual or potential adverse effects, or contribution to pill burden, might outweigh benefit) | Impact on health outcomes |
| certainty of estimate of effects (e.g. where benefit for continued use of the medication is uncertain) | Availability of low certainty evidence |
| patient preference and values (e.g. is it acceptable to patients?) | Interest at consumer level; Acceptability |
| feasibility (e.g. from both patient and physician perspective) and cost (e.g. cost savings to the system or patient) | Feasibility of intervention implementation, Economic impact, Impact on healthcare system |
| need for guidance in relation to both stopping the medication and managing the impact of stopping the medication | Absence of guidance |
| uncertainty of benefit in the elderly | Availability of low certainty evidence |
| high risk of harms in the elderly | Impact on health outcomes |
| availability of suitable alternatives | Impact on health outcomes |
| potentially high impact of a deprescribing guideline for the elderly | Judged by authors as irrelevant |
| feasibility for guideline development (i.e. an adequate amount of literature to create an evidence-based guideline) | Availability of evidence |
| van der Sanden, 2002 | |
| *No explicit criteria used* |  |
| Borgonjen, 2015 (13 criteria) | |
| Relevancy for the stakeholders | Interest at health professional, consumer and national levels |
| Unwanted variation in practice | Practice variation |
| The potential to improve quality of life/lessen burden of illness | Impact on health outcomes |
| The applicability/implementability in practice | Feasibility of intervention implementation |
| The availability of scientific evidence to underpin recommendations | Availability of evidence |
| The need/demand of a guideline across stakeholders | Interest at health professional, consumer and national levels |
| The prevalence/incidence/morbidity of a disease | Health burden |
| The potential to improve the quality of public healthcare | Impact on health outcomes; Impact on health system |
| The potential to reduce costs on the macroeconomic level | Economic impact |
| The mortality of a disease | Health burden |
| political relevance of a topic | Interest at health professional, consumer and national levels |
| the role of the dermatologist in a multidisciplinary topic | Interest at health professional level |
| guidance in topics with many offlabel or unregistered treatments | Availability of low certainty evidence |
| van der Veer, 2016 (characterizing high-priority topics as decisions or practices:) (4 criteria) | |
| common in clinical practice | Health burden |
| strongly affected patient outcomes | Impact on health outcomes |
| uncertainty or disagreement around optimal care | Uncertainty or controversy about best practice |
| may be associated with substantial risk of patient harm (e.g., adverse events) or high costs for the healthcare system | Impact on health outcomes; Burden on healthcare system |
| Jo, 2015 (4 criteria) | |
| prevalence rate | Health burden |
| mortality | Health burden |
| medical expenses | Economic burden |
| disability-adjusted life years (DALYs) | Health burden, Economic burden |
| Prioritizing update of guidelines | |
| Becker, 2018 (8 criteria) | |
| clinical relevance of existing guideline sections | |
| frequency of clinical problem | Health burden |
| variation of treatment practices | Practice variation |
| controversial discussion on clinical problems | Uncertainty or controversy about best practice |
| need for change of recommendations | Unsatisfactory guidance |
| lacking of relevant recommendations | Absence of guidance |
| new evidence of existing guideline sections | |
| new evidence that warrants a change of recommendation | Potential for changing existing guidance |
| new evidence that warrants a change of grade of recommendation | Potential for changing existing guidance |
| new evidence that warrants supplemental recommendations | Potential for changing existing guidance |
| Agbassi, 2014 (10 criteria) | |
| Current relevance of the CPG to cancer care: Is the document still relevant (clinically or to the cancer care system as a whole in some way)? | Potential for changing existing guidance, Health burden |
| Timing of assessment: Should full assessment and review of this document be deferred until next year?  Consider YES if:  - The document is less than three years old, and there is no reason to doubt the recommendations  - The document is between three and five years old, and a justification can be provided as to why the recommendations can be considered trustworthy for another year | Unsatisfactory guidance |
| Need for updated literature search and appropriateness of questions and search criteria: Do the questions and search criteria as they are in the document address current needs, such that an updated literature search would be useful and identify relevant evidence?  Consider NO if:  - The standard of care has shifted significantly since the last version of the document, such that the questions only address the topic in part - There are new, significant options (for treatment, diagnosis, etc.) available that are not covered by the current questions, such that new questions would need to be added to the document  - In general, if you believe that for the document to still be useful it will have to substantially be rewritten - The document has been repeatedly deferred, and is now older than five years | Potential for changing existing guidance |
| Impact of the CPG on access to care Does the document have an impact on access to care (that is, are decisions about access or payment for care made by the Ministry, CCO, or other organizations based on the recommendations in this document)? Consider YES if:  - Ministry funding decisions have been, are, or will be made on the basis of this document  - An indication for a chemotherapy regimen was funded, or rejected, based on the document - Case by case review or out of country requests are known to be decided based on the document  - Funding for some screening, diagnostic, staging or treatment procedure was or is determined | Impact on equity/access |
| Availability of new evidence capable of invalidating the existing recommendations Is there known evidence that has been published since this document’s last literature search (see above) that would result in significant changes to the recommendations? | Potential for changing existing guidance |
| Risk of leaving the document publicly available while undergoing Document Assessment and Review Should this document be taken off the website while it awaits full review, or can it be left there with an “IN REVIEW” watermark?  Consider YES if:  - If followed, even in error, the recommendations have the potential to cause harm to patients | Judged by authors as irrelevant |
| If newly identified evidence supports or contradicts current recommendations Does any of the newly identified evidence, on initial review, contradict the current recommendations, such that the current recommendations may cause harm or lead to unnecessary or improper treatment if followed? | Unsatisfactory guidance; Potential for changing existing guidance |
| if the current recommendations cover all relevant subjects addressed by the new evidence, and if strong evidence that may change the current recommendations is expected to be published in the near future On initial review,  - Does the newly identified evidence support the existing recommendations?  - Do the current recommendations cover all relevant subjects addressed by the evidence, such that no new recommendations are necessary? | Potential for changing existing guidance |
| Is there a good reason (e.g., new stronger evidence will be published soon, changes to current recommendations are trivial or address very limited situations) to postpone updating the guideline? | Availability of evidence |
| Do the PEBC and the DSG/GDG responsible for this document have the resources available to write a full update of this document within the next year? | Availability of resources |
| van der Veer, 2015 (7 criteria) | |
| *criteria used with clinicians* | |
| highest priority to topics for which having a robust evidence base is most relevant within your clinical practice | Interest at health professional level |
| highest priority to topics referring to clinical situations, procedures or decisions regarding vascular access care in haemodialysis patients that are common in your daily practice | Health burden |
| highest priority to topics referring to clinical situations, procedures or decisions regarding vascular access care in haemodialysis patients that strongly affect the outcomes of your patients (e.g., quality of life, survival) | Impact on health outcomes |
| highest priority to topics for which there is uncertainty or disagreement around optimal care | Uncertainty or controversy about best practice |
| highest priority to topics that are associated with high costs for your patient (side effects, adverse events) or for the healthcare system (resource use) | Economic burden; Burden on healthcare system |
| *criteria used with patients* | |
| How strongly do I prefer one option over the others? | Interest at the consumer level |
| How much effort would it take my doctor to convince me that the other options are better? | Judged by authors as irrelevant |
