# Supplementary material for: The implementation of prioritization exercises in the development and update of health practice guidelines: A scoping review
Source: PLoS One. 2020 Mar 20;15(3):e0229249. doi: 10.1371/journal.pone.0229249 (PMC7083273; doi:10.1371/journal.pone.0229249)
Supplement: Attachment — Submitted filename: PONE-D-19-24700_comments.docx [file pone.0229249.s006.docx]

**Manuscript Number: PONE-D-19-24700**

**Manuscript Title: The implementation of prioritization exercises in the development and update of health practice guidelines: a systematic review**

This study sought to systematically review the use of prioritisation processes for developing, updating, or adapting clinical practice guidelines in health care. Twelve eligible studies are included and described, of which all focussed on prioritising broad clinical topics. There was variation in the prioritisation exercises on various domains, including stakeholder involvement, steps of prioritisation addressed, and criteria used to prioritise topics.

This is a comprehensive and rigorous review with important implications for future research and guideline development. The rationale, objectives, and potential value of the review are clearly described. In general, the manuscript is very well-written, particularly the introduction, methods, and results. The manuscript and supplementary files are well-organised and appropriately detailed. The authors should be commended on their clear and comprehensive reporting of methods and results. Overall, this review offers several important observations about prioritisation processes, including potential deficiencies, which can assist to improve and standardise such initiatives for researchers and guideline developers.

I have a few comments and suggestions, which focus mainly on the Discussion.

Main comments:

- I felt the 'summary of findings' had omitted some interesting and important results of the review that would be worth briefly summarising in that section of the manuscript. Specifically, the dominant criteria used to prioritise topics (i.e. health burden and impact on health outcomes) and stakeholder involvement (i.e. this this was largely focussed in care providers). The latter is particularly important to mention in the summary given (diverse) stakeholder involvement is critical for maximising relevance and uptake of guidelines, as stated in the introduction and later in the discussion.
- It was striking that only one of the prioritisation exercises engaged patients or their representatives. This is a clear gap in the reviewed prioritisation exercises and it is worth exploring in the discussion the potential reasons why this might be the case. For example, it may be that research compliance issues and/or funding and resource limitations makes patient-engagement more difficult? These sorts of potential barriers (and their possible solutions) could be mentioned in paragraph 3 of the interpretation of findings (p. 30).
- Paragraph 4 of the interpretation of results (p. 30) could be expanded. It is noted that all studies adopted an online approach to engagement, likely for practical and financial reasons. It is worth mentioning the other methodologies available for priority-setting exercises, that have been potentially under-utilised (e.g. workshops and face-to-face meetings). Such methodologies may be more accessible for certain stakeholder types, and may also yield different priorities…
- As a whole, the interpretation of results felt somewhat brief. I felt it could be improved by drawing out a few more of the review's salient findings, for discussion and critical appraisal. For example, only 3 studies reported on the development of ethical principles to guide the conduct of the exercise; only 3 reported a plan for dissemination; and none included equity relevance of the condition. Do the authors see these as important gaps in current practice that should be addressed in future priority setting exercises?
- The authors state that having not searched the grey literature was a limitation of their review. It was not clear to me what was meant by "Nonetheless, these would require a different search strategy and would not reflect real life exercises.". Was the intention to say that prioritisation exercises would not be published in grey literature? Please clarify in the manuscript.
- I think the findings of this review will also be of benefit to researchers who may design and conduct priority setting activities in future. It is worth adding 'researchers' explicitly to the list given in the opening paragraph of the 'implications for practice' (p. 32).
- As the authors state under the implications for practice, "the decision on whether and how to conduct a prioritization exercise should be tailored to the needs of relevant stakeholders and to the available resources, including time and funding" (p. 32). Indeed, this may explain some of the variation in the prioritisation exercises reviewed. It is worth stating this in the discussion.
- I think it is important to (re)state in the conclusion the importance of diverse stakeholder involvement, particularly with regard to patients and their representatives, which the review has highlighted as a significant gap.

Minor comments:

- The % symbol is missing from the data label row in Table 4.
- There is an 's' missing from 'stakeholder' in the second paragraph under Stakeholder involvement (p. 20).
